# Characterization of Microfragmented Adipose Tissue Architecture, Mesenchymal Stromal Cell Content and Release of Paracrine Mediators

**DOI:** 10.3390/jcm11082231

**Published:** 2022-04-15

**Authors:** Enrico Ragni, Marco Viganò, Enrica Torretta, Carlotta Perucca Orfei, Alessandra Colombini, Carlo Tremolada, Cecilia Gelfi, Laura de Girolamo

**Affiliations:** 1 Laboratorio di Biotecnologie Applicate all’Ortopedia, IRCCS Istituto Ortopedico Galeazzi, Via R. Galeazzi 4, I-20161 Milano, Italy; enrico.ragni@grupposandonato.it (E.R.); marco.vigano@grupposandonato.it (M.V.); carlotta.perucca@grupposandonato.it (C.P.O.); alessandra.colombini@grupposandonato.it (A.C.); 2 Laboratorio di Proteomica e Scienze Separative, IRCCS Istituto Ortopedico Galeazzi, Via R. Galeazzi 4, I-20161 Milan, Italy; enrica.torretta@unimi.it (E.T.); cecilia.gelfi@unimi.it (C.G.); 3 Image Regenerative Clinic, Via Mascagni 14, I-20122 Milan, Italy; carlo.tremolada@gmail.com; 4 Department of Biomedical Sciences for Health, University of Milan, Via Fratelli Cervi 93, I-20054 Segrate, Italy

**Keywords:** osteoarthritis, regenerative medicine, adipose tissue, lipoaspirate, microfragmentation, mesenchymal stromal cells, proteomics, extracellular vesicles, miRNAs

## Abstract

The use of microfragmented adipose tissue (µFAT) for the treatment of musculoskeletal disorders, especially osteoarthritis (OA), is gaining popularity, following positive results reported in recent case series and clinical trials. Although these outcomes were postulated to rely on paracrine signals, to date, a thorough fingerprint of released molecules is largely missing. The purpose of this study was to first characterize both structure and cell content of unprocessed lipoaspirate (LA) and µFAT, and further identify and frame the array of signaling factors in the context of OA disease, by means of high throughput qRT-PCR for extracellular-vesicle (EV) embedded miRNAs and proteomics for tissue and secreted factors. Cell count showed reduction of blood cells in µFAT, confirmed by histological and flow cytometry analyses, that also showed a conserved presence of structural, endothelial and stromal components and pericytes. In the secretome, 376 and 381 EV-miRNAs in LA and µFAT, respectively, were identified. In particular, most abundant and µFAT upregulated EV-miRNAs were mainly recapitulating those already reported as ASC-EVs-specific, with crucial roles in cartilage protection and M2 macrophage polarization, while only a scarce presence of those related to blood cells emerged. Furthermore, secretome proteomic analysis revealed reduction in µFAT of acute phase factors driving OA progression. Taken together, these results suggest that processing of LA into µFAT allows for removal of blood elements and maintenance of tissue structure and stromal cell populations, and possibly the increase of OA-protective molecular features. Thus, microfragmentation represents a safe and efficient method for the application of adipose tissue properties in the frame of musculoskeletal disorders.

## 1. Introduction

Osteoarthritis (OA) is one of the most widespread causes of disability worldwide, affecting 7% of the global population and responsible for 2% of the total global years lived with disability [1]. OA has a dramatic impact on patients’ quality of life and is characterized by joint pain, swelling and loss of function. In particular, wear of subchondral bone and articular cartilage, osteophyte formation, inflammation and loss of normal joint function, are pathognomonic factors in the early and more advanced stages of OA [2]. Conservative non-pharmacologic and pharmacologic management, although effective to some extent, provide only temporary benefits and are often associated with side effects [3]. This situation has led to a search for new medications based on “orthobiologics”, able to both relieve pain and possibly counteract, or delay, joint tissue degeneration.

In this category, platelet-rich plasma (PRP)- and mesenchymal stromal cell (MSC)-based products have rapidly gained popularity. Despite positive results in improving articular knee function and pain management [4], magnetic resonance imaging (MRI) studies showed controversial results, with both investigations not confirming improvement in tissue regeneration and cartilage thickness [5,6], and reports indicating increased content of cartilage glycosaminoglycans in specific areas of the treated knee joint [7]. MSCs work as active trophic mediators by sensing the environment [8] and secreting both soluble factors and extracellular vesicle (EV)-embedded regulatory molecules, such as miRNAs, collectively defining the “secretome” [9]. These factors inhibit inflammation, tissue fibrosis and apoptosis, and stimulate activation of tissue-intrinsic progenitor cells [10]. The multipotency of adipose tissue resident-MSC (ASC), especially toward the chondrogenic lineage [11], the abundance of this tissue in the body and its easier collection, with respect to other MSC-containing tissues, such as bone marrow, emphasized its potential as medication for treating joint degeneration [12]. Within the different types of adipose tissue-derived products [12], microfragmented adipose tissue (μFAT) is obtained using mild mechanical forces to wash away pro-inflammatory oil and blood residues, without the use of enzymes, additives or centrifugation. A crucial aspect of fragmentation is the preservation of tissue microarchitecture [13], consisting of small lobules of the stromal vascular niche, facilitating greater trophic and regenerative qualities than adipose tissue harvested according to the “standard” technique [14]. This is due to the increased surface/volume ratio making active cells, such as MSCs and pericytes, more easily available [15]. In fact, under inflammatory conditions, or when there is tissue damage, pericytes can detach from the capillary wall and gradually convert into, and implement, a pool of medicinal and activated MSCs [16]. Consistently, recent studies have shown significant clinical improvement after μFAT injection for the treatment of knee OA. More clinical trials are currently undergoing [17].

Despite these promising results, the exact mechanism of action of μFAT, and the way it may interact with the osteoarthritic microenvironment, is not fully understood and is supposed to rely on the release of soluble factors and EVs. The aim of this work was to characterize μFAT architecture in terms of structure and the most relevant stromal cell type composition, and compare with unprocessed lipoaspirate (LA) obtained from the same patients. For the first time, to our knowledge, we performed a deep characterization of both tissue and soluble factors in both products (μFAT and donor-matched LA) via proteomics and released EVs and embedded miRNAs via high-throughput qRT-PCR.

## 2. Materials and Methods

### 2.1. Adipose Tissue Harvesting and Processing

Adipose tissue was collected from 7 donors (3 males, 4 females, mean age 44 ± 6) during liposuction with Coleman’s technique [18]. Part of the lipoaspirate tissue (LA) was processed to obtain microfragmented adipose tissue (μFAT), according to the manufacturer’s instructions, previously described [16] (Lipogems^®^ International, Milan, Italy). Both Lipogems 120TM and 240TM devices were used to highlight possible differences, in term of features of the final products. First, reduction of lipoaspirate clusters was performed by pushing the lipoaspirate through a filter into the device where five stainless steel beads facilitated the obtaining of an emulsion of oil, blood, and saline. The suspension was washed against density by means of a current of saline moved by gravity in the wasting bag. After washing, further adipose cluster reduction was performed through a second filter and µFAT was decanted by gravity in order to remove excessive saline solution. 

### 2.2. Histology and Immunohistochemistry

Adipose tissue samples were fixed in 10% neutral buffered formalin (Sigma-Aldrich, St. Louis, MO, USA) for 24 h at RT, embedded in paraffin and sectioned at 4 μm. Hematoxylin and Eosin (H&E) staining was performed to evaluate the structure and morphology of the samples. The presence and localization of CD31, CD146 and CD90 markers were determined by immunohistochemistry staining. Briefly, sections were incubated with 1X Dewax and HIER Buffer H solution (Thermo Fisher Scientific, Madison, WI, USA) at 60 °C overnight for antigen retrieval, permeabilized with 0.1% Triton X-100 in PBS for 20 min at RT, and incubated with blocking buffer 3% BSA in PBS for 1 h at RT. Samples were then incubated at 4 °C overnight with the following primary antibodies: rabbit monoclonal to CD31 (1:2000, ab182981; Abcam, Cambridge, MA, USA), mouse monoclonal to CD90 (1:500, sc53456; Santa Cruz Biotechnology, Santa Cruz, CA, USA) and mouse monoclonal to CD146 (1:500, sc374556; Santa Cruz, CA, USA) antibodies. Sections were then washed with PBS buffer and incubated for 1h with anti-rabbit IgG, (H + L) raised in goat, biotinylated (1:200, VC-BA-1000-MM15; Vector Laboratories, Burlingame, CA, USA) or with anti-mouse IgG, (H + L) raised in horse, biotinylated (1:200, VC-BA-2000-MM15, Vector Laboratories) secondary antibodies. At the end of the immunohistochemistry staining, microscope images were acquired by means of an Olympus IX71 (Olympus, Hamburg, Germany) inverted microscope.

### 2.3. Cell Count and Viability

All samples were incubated with collagenase type I 0.075% w/v (Worthington Biochemical Corporation, Lakewood, NJ, USA) at 37 °C for 45 min in order to isolate the stromal vascular fraction (SVF) [19]. Washing was avoided at all stages to prevent removal of blood elements during this phase. Cells were then centrifuged and passed through a 100 µm filter to remove aggregates, before resuspension in phosphate buffer solution (PBS). Complete blood count was performed on these suspensions by the diagnostic laboratory of the Institute using an hematology analyzer Sysmex XN-2000 (Sysmex, Kobe, Japan) and, at the same time, cell count and viability were assessed with a Nucleocounter NC-3000 and cell viability staining (solution 17) (Chemometech, Allerod, Denmark) following manufacturer’s instructions [20].

### 2.4. Flow Cytometry

Immunophenotype analysis: Freshly isolated SVF (around 2 mL) was incubated for 10 min at RT with 10 volumes of Red Blood Cell Lysis Solution (Miltenyi Biotec, Bergisch Gladbach, Germany) to remove erythrocytes. After centrifugation, the pellet was washed once with 10 mL FACS buffer and eventually the pellet was suspended in 200 µL FACS buffer and divided into 2 aliquots. To identify the specific subpopulations [21], one aliquot was analyzed through combination of the antibodies CD45-PE-Vio770 (REA747, Miltenyi), CD31-APC (WM59, Biolegend, San Diego, CA, USA), CD34-PE (AC136, Miltenyi), CD90-FITC (REA897, Miltenyi), CD105-PerCP-Vio700 (REA794, Miltenyi), CD146-APC/Fire (P1H12, Biolegend). Cells were incubated for 30 min at 4 °C in the dark, following the manufacturer’s instructions. The second aliquot of cells was processed in the same way without primary antibodies and used as a negative control for staining. Then, cells were washed with 1 mL FACS buffer and the pellet suspended in 250 µL FACS buffer. All samples were analyzed using a flow cytometer (Cytoflex, Beckman Coulter Inc., Fullerton, CA, USA) acquiring at least 30,000 events.

Senescence analysis: For SVF obtained from LA, a 1:50 dilution was prepared in PBS. SVF (diluted or undiluted, 100 µL) was split into 2 aliquots. One aliquot was left unstained and used as the negative control, while the other was stained with CD45-PE-Vio770 (REA747, Miltenyi) and CD235a-PE (REA175, Miltenyi) antibodies for 30 min at 4 °C in the dark, following the manufacturer’s instructions. Then, both aliquots were washed with 1 mL Fluorescein di-β-D-galactopyranoside (FDG) buffer (PBS, 10 mM Hepes, 5% FBS, pH 7.3). The control aliquot was supplemented with 150 µL FDG buffer while the stained aliquot was divided into two 35 µL aliquots. One aliquot was supplemented with 35 µL FDG buffer as a negative control for FDG and the other with 35 µL 2 mM FDG, and both were incubated for 5 min at 37 °C in the dark. Then, both aliquots were supplemented with 80 µL ice-cold FDG buffer and all three samples (Neg, CD45/CD235a stained, and CD45/CD235a/FDG stained) were analyzed using a flow cytometer (Cytoflex, Beckman) acquiring at least 30,000 events.

### 2.5. ASCs Isolation and Differentiation

Following protocol for cell isolation as per Section 2.3, pellets were eventually suspended in DMEM + 10% FBS before seeding at 5 × 10^3^ cells/cm^2^ (37 °C, 5% CO_2_, 95% humidity) and ASCs were selected by plastic adherence. For adipogenic differentiation, a repeated pulsed protocol, consisting of 3 days in an adipogenic induction medium (control medium supplemented with 1 μM dexamethasone, 10 μg/mL insulin, 500 μM 3-iso-butyl-1-methylxanthine and 200 μM indomethacin), followed by three days in a maintenance medium (control medium supplemented with 10 μg/mL insulin) was conducted, for a total of 21 days. Lipid vacuoles were visualized with Oil Red O (Sigma-Aldrich, 3 parts of a 0.5% stock solution in isopropanol and 2 parts of distilled water). For osteogenic differentiation, osteogenic medium (control medium supplemented with 10 mM glycerol-2-phosphate, 10 nM dexamethasone, 150 μM l-ascorbic acid-2-phosphate and 10 nM cholecalciferol) was used for 21 days. Extracellular calcified matrix deposition was visualized with Alizarin Red S (Sigma-Aldrich, 2 g/100 mL in distilled water). For chondrogenic differentiation, chondrogenic medium (control medium supplemented with 100 U/mL penicillin, 100 μg/mL streptomycin, 0.29 mg/mL L-glutamine, 1 mM sodium pyruvate, 1.25 mg/mL human serum albumin, 1% ITS+1, 0.1 μM dexamethasone, 0.1 mM L-ascorbic acid-2-phosphate, and 10 ng/mL TGF-β1) was used for 21 days. Chondrogenic proteins were visualized with Alcian blue (Sigma-Aldrich, 1 g/l in 0.1 M HCl).

### 2.6. Secretome Collection

LA or μFAT (around 6 mL each) were supplemented with DMEM in a 1:1 volume ratio, and incubated for 24 h at 37 °C in a humidified atmosphere at 5% CO_2_. Then, the total volume was centrifuged at 376× *g* for 5 min at 4 °C and the liquid phase collected with a syringe. The liquid phase was filtered with a 100 µm cell strainer and centrifuged at 376× *g* for 5 min at 4 °C. The supernatant was sequentially centrifuged at 1000, 2000 and 4000 (twice)× *g* for 10 min at 4 °C. Eventually, the supernatant was filtered through a 1.2 µm filter and the cleared sample collected. The sample was further divided into three aliquots: 3 mL for EV retrieval and EV-miRNA detection, 2 mL for proteomic analysis, and the remaining for EV size distribution analysis. Aliquots were stored at −80 °C until use.

### 2.7. Proteomic Analysis

Tissue samples were suspended in lysis buffer (1% sodium deoxycholate, 8 M Urea, 50 mM ammonium bicarbonate, 5 mM DTT, 1 mM phenylmethylsulfonyl fluoride (PMSF)) and sonicated. Supernatants were first concentrated with Amicon 5 kDa (Sigma-Aldrich) and mixed with 1% SDS and 5 mM DTT, prior to sonication.

Protein concentration was determined by 2D Quant Kit (GE Healthcare, Buckinghamshire, UK). Following the FASP (Filter-Aided Sample Preparation) protocol [22], one-hundred μg of proteins from each sample were placed in 30 kDa filters (Sigma-Aldrich) for in-tube reduction (100 mM DTT in 50 mM ammonium bicarbonate), alkylation (0.05 M iodoacetamide in 8 M urea in 0.1 M Tris/HCl pH 8.5) and digestion with 1:50 (*w*/*w*) Trypsin Gold, MS grade (Promega, Madison, WI, USA) for 16 h at 37 °C.

The obtained peptides were subjected to LC-ESI-MS/MS shotgun analysis, performed on a Dionex UltiMate 3000 HPLC System with an Easy Spray PepMap RSLC C18 column (150 mm, internal diameter of 75 μm; Thermo Fisher Scientific) with the following gradient: 5% acetonitrile (ACN) in 0.1% formic acid for 10 min, 5–35% ACN in 0.1% formic acid for 79 min, 35–60% ACN in 0.1% formic for 40 min, 60–100% ACN for 1 min, 100% ACN for 10 min at a flow rate of 0.3 μL/min. The eluate was electrosprayed into an Orbitrap Tribrid Fusion (Thermo Fisher Scientific) through a nano-electrospray ion source (Thermo Fisher Scientific). The LTQ-Orbitrap was operated in positive mode in data-dependent acquisition mode to automatically alternate between a full scan (350–2000 *m*/*z*) in the Orbitrap (at resolution 60,000, AGC target 1,000,000) and subsequent CID MS/MS in the linear ion trap of the 20 most intense peaks from full scan (normalized collision energy of 35%, 10 ms activation). Isolation window: 3 Da, unassigned charge states: rejected, charge state 1: rejected, charge states 2+, 3+, 4+: not rejected; dynamic exclusion enabled (60 s, exclusion list size: 200). Mass spectra were analyzed using MaxQuant software [23] (version 1.6.3.3). The initial maximum allowed mass deviation was set to 6 ppm for monoisotopic precursor ions and 0.5 Da for MS/MS peaks. Enzyme specificity was set to trypsin/P, and a maximum of two missed cleavages was allowed. Carbamidomethylation was set as a fixed modification, while N-terminal acetylation and methionine oxidation were set as variable modifications. The spectra were searched by the Andromeda search engine against the Homo Sapiens Uniprot sequence database (release 15 January 2020) [24]. Protein identification required at least one unique or razor peptide per protein group. Quantification in MaxQuant was performed using the built-in XIC-based label-free quantification (LFQ) algorithm using fast LFQ [25]. The required false positive rate (FDR) was set to 1% at the peptide, 1% at the protein and 1% at the site-modification level, and the minimum required peptide length was set to 7 amino acids.

Bioinformatics analysis was carried out by Ingenuity Pathway Analysis (IPA^®^) (QIAGEN, Hilden, Germany). The quantitative protein data were imported into IPA software to identify canonical pathways and diseases and biofunctions most strongly associated with the protein lists. The software works by experimental expression data on networks constructed from published interactions by giving a score (z-score), indicating up-regulation (z-score > 2) or down-regulation (z-score < 2).

### 2.8. Nanoparticle Tracking Analysis (NTA)

Cleared samples (1:20 PBS diluted) were visualized by the Nanosight LM10-HS system (NanoSight Ltd., Amesbury, UK), with five recordings of 30 s for each sample. NTA software was used to analyze the data and provided both the concentration measurements and the high-resolution particle size distribution profiles.

### 2.9. EV-Associated MicroRNAs (miRNAs) High Throughput Analysis

Secretome aliquots (3 mL) were diluted to 10 mL with triple 0.2 µm filtered PBS and centrifuged for 9 h at 100,000× g at 4 °C in a 70Ti rotor (Beckman). The pellet was dissolved in Trizol reagent (Thermo Fisher Scientific). MiRNeasy and RNeasy CleanUp Kits were sequentially used to isolate RNA enriched in small molecules (<200 nt) (Qiagen). Six pg of a non-human synthetic miRNA (Arabidopsis thaliana ath-miR-159a) were added before extraction to each sample as a spike-in to validate the technical variability during the RNA isolation procedure, during the following reactions and to equalize A and B panels of the OpenArray^®^ platform (Thermo Fisher Scientific). CDNAs were prepared by standard reverse transcription protocol, and preamplification performed with A and B independent kits, followed by real-time RT-PCR analysis with the QuantStudio™ 12 K Flex OpenArray^®^ Platform (QS12KFlex) [26]. The Expression Suite Software (Thermo Fisher Scientific) was used to process miRNA expression data from the A and B miRNA panels, covering 754 human miRNAs (Sanger miRBase v21). C_RT_ values > 28 were considered as absence of amplification. The global mean of shared EV-miRNAs was used to normalize expression data [27]. EV-miRNA abundance between samples was determined using relative quantification 2^−ΔCRT^.

### 2.10. Transmission Electron Microscopy (TEM)

After ultra-centrifugation, EV pellets were suspended in PBS (100 μL per initial 3 mL supernatants). 5 μL were absorbed for 10 min at RT on formvar carbon-coated grids and excess liquid was removed with filter paper. Negative staining was done with 2% uranyl acetate aqueous suspension for 10 min and excess liquid was removed with filter paper. The grid was dried at RT. Samples were examined with a TALOS L120C transmission electron microscope (Thermo Fisher Scientific, Waltham, MA, USA) at 120 kV.

### 2.11. Principal Component Analysis (PCA) and Hierarchical Clustering

PCA and heat map plots were generated with ClustVis package (https://biit.cs.ut.ee/clustvis/, accessed on 30 September 2021) [28]. Clustering options for the heat map were distance for rows, correlation, method for rows, average and tree ordering for rows, tightest cluster first.

### 2.12. Statistical Analysis

Statistical Analyses were performed using Graphpad Prism v5.0 (Prism Software Inc., La Jolla, CA, USA). The Shapiro-Wilk test was used to assess normal distribution of data. On the basis of these results, the comparison among groups was performed using one-way ANOVA for repeated measures, with Bonferroni’s post-test, or Friedman’s test, with Dunns’ post-test. For proteomics, Student *t*-test with post-hoc test Permutation based FDR < 0.05, conducted using Perseus software (version 1.6.1.3). Only proteins present and quantified in at least 80% of the technical and biological repeats were considered as positively identified in a sample and used for statistical analyses.

## 3. Results

Due to identical results obtained with the two devices under study (Lipogems120^TM^ and 240TM), and for ease of presentation, only data obtained with 120TM are herein presented.

### 3.1. Tissue Integrity Is Maintained in μFAT

**Figure 1:**
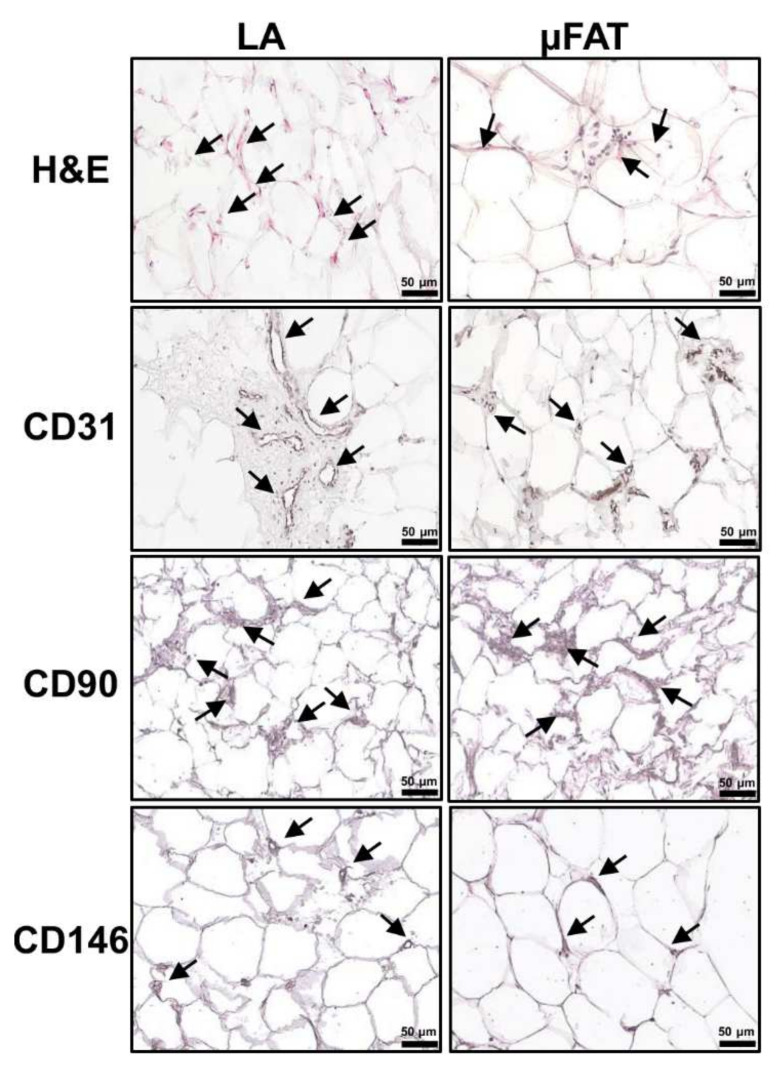
Immunohistochemical localization of vascular, stromal and perycitic markers in lipoaspirate (LA) and microfragmented (µFAT) tissues. Human adipose tissues were examined by immunohistochemistry for the expression of α-CD31 (vascular endothelium), α-CD90 (stromal cells) and α-CD146 (perycites). Positively stained structures are marked with arrows. Control specimen stained with H&E (without antibody) is shown on top. Representative images for each tissue type are shown.

Histological investigations showed a lower red blood cell presence in the μFAT compared to unprocessed LA, as emerged in the H and E staining (Figure 1). Blood vessels were identified in all the samples by CD31 labeling, although in µFAT their size appeared to be reduced. CD90 positivity given by stromal cells was comparable, and spread within the architecture of the tissue. In addition, few pericytes (CD146^+^) embraced small vessels, without differences among µFAT and LA.

### 3.2. Microfragmentation Allows for Removal of Blood Contamination

**Figure 2:**
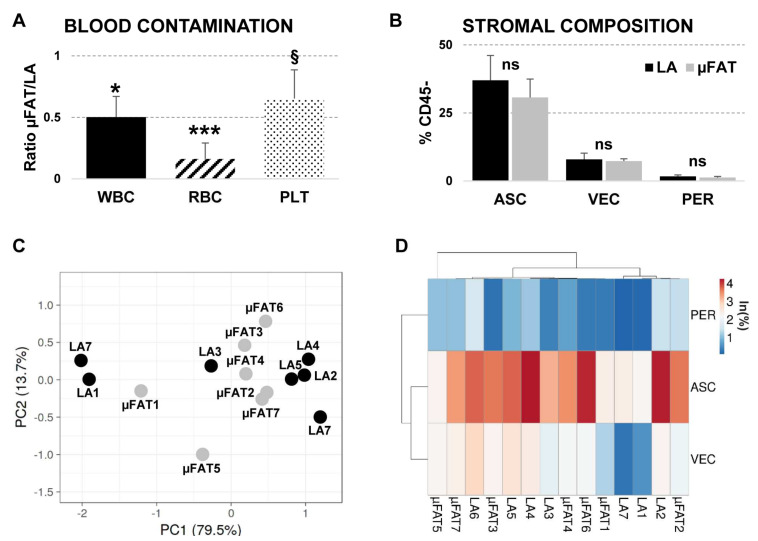
Cell composition of lipoaspirate (LA) and microfragmented (µFAT) tissues. (**A**) Blood elements as white blood cells (WBC), red blood cells (RBC) and platelets (PLT) were counted with an haematology analyzer. N = 7. § for *p*-value ≤ 0.1, * *p*-value ≤ 0.05 and *** *p*-value ≤ 0.001. (**B**) Adipose stromal cells (ASC, CD31^−^CD34^+^CD90^+^CD105^Low^CD146^−^), vascular endothelial cells (VEC, CD31^+^CD34^+^CD90^+^CD105^−/L^CD146^+^) and pericytes (PER, CD31^−^CD34^−^CD90^+^CD105^−^CD146^+^) were detected by flow cytometry on the CD45^−^ population. N = 7. ns for not significant *p*-value. (**C**,**D**) Principal component analysis (PC = Principal Component in panel C) and hierarchical clustering obtained with % values of the different CD45^−^ populations (ln transformed) identified by flow cytometry and presented in panel B. Clustering options for heat map were distance for rows—correlation, method for rows—average and tree ordering for rows—tightest cluster first.

Microfragmentation resulted in a loss of blood-related cells (absolute count WBC (×10^3^/µL) 1.4 ± 0.6 vs. 0.4 ± 0.1, RBC (×10^3^/µL) 177.1 ± 70.0 vs. 7.1 ± 2.9, PLT (×10^3^/µL) 12.4 ± 6.4 vs. 3.0 ± 0.6, LA vs. µFAT mean ± SEM)), with µFAT/LA single ratios shown in Figure 2A and the overall µFAT/LA ratio of 0.44 ± 0.12 (LA set to 1, *p*-value 0.040). These data were confirmed by flow cytometry analyses for leukocytes (CD45^+^) and erythrocytes (CD45^−^CD235a^+^), showing a 79% (*p*-value < 0.001) and 76% (*p*-value 0.002) reduction, respectively. Eventually, viable cells reduced from 67% ± 6 to 49% ± 8 in µFAT with respect to unprocessed LA (*p*-value 0.011).

### 3.3. Population Heterogeneity Is Maintained in μFAT

Both LA and μFAT CD45^−^ stromal populations were characterized by flow cytometry (Supplementary Figure S1) for the presence of vascular endothelial cells (CD45^−^CD31^+^CD34^+^CD90^+^CD105^L^CD146^+^), ASCs (CD45^−^CD31^−^CD34^+^CD90^+^CD105^−/L^CD146^−^) and pericytes (CD45^−^CD31^−^CD34^−^CD90^+^CD105^−^CD146^+^) [21]. No significant differences emerged (*p*-values always ns) (Figure 2B). Adipose-derived stromal population consistently resulted in around 30% of the total events, complying with the guidelines of the International Federation for Adipose Therapeutics and Science (IFATS) and the International Society for Cellular Therapy (ISCT), that defined the stromal population falling into 15–30% of the SVF [29]. One third of the CD45^−^ population fell within the ASC phenotype (40.0 ± 9.1% vs. 30.7 ± 6.8%, LA vs. µFAT mean ± SEM), while vascular endothelial cells and pericytes accounted for 7.9 ± 2.3% vs. 7.3 ± 0.9% and 1.6 ± 0.6% vs. 1.2 ± 0.4% (LA vs. µFAT mean ± SEM), respectively. Principal Component Analysis (PCA) associated with Hierarchical Clustering evaluated the effect of the processing on the ratios of the different stromal cell populations with respect to the original LA composition, and no distinct clusters emerged; suggesting that microfragmentation does not select for specific stromal populations (Figure 2C,D). Isolated ASCs from both LA and µFAT were able to undergo osteogenic, adipogenic and chondrogenic differentiation (Supplementary Figure S2).

Finally, the percentage of senescent cells in the CD45^−^ population was similar before and after tissue processing, with very low values (≤5%, data not shown).

### 3.4. Microfragmentation Modulates the Protein Fingerprint

**Figure 3:**
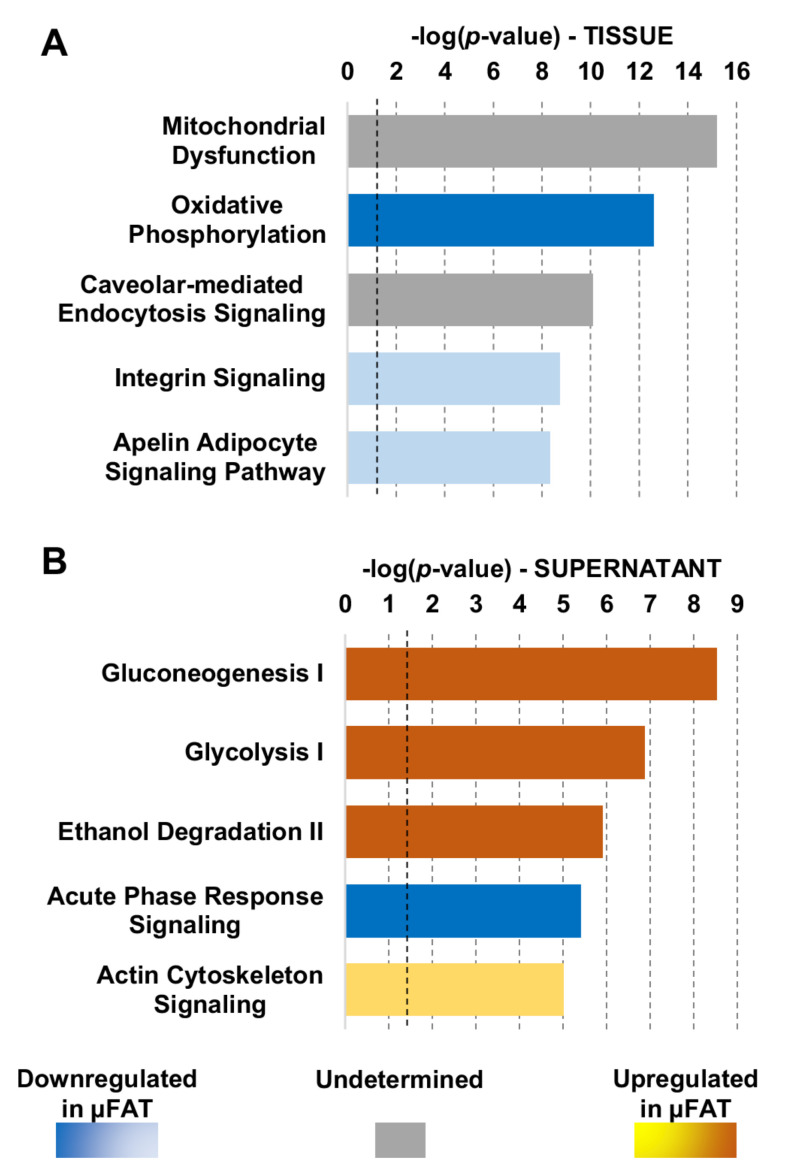
Dysregulated processes in protein tissues and supernatants after microfragmentation. (**A**) The top five most dysregulated pathways identified by IPA analysis in the µFAT vs. LA tissues accordingly to *p*-value. Black dotted line indicates the threshold for *p*-value significance. Blue colour indicates a negative z-score and pathway inhibition in µFAT, while grey colour indicates that IPA software was not able to assign a clear regulation regarding activation or inhibition. (**B**) The top five most dysregulated pathways in the µFAT vs. LA supernatants accordingly to *p*-value. Black dotted line indicates the threshold for *p*-value significance. Blue colour indicates a negative z-score and pathway inhibition in µFAT, while array of orange colour indicates a positive z-score and therefore pathway activation, with darker colour meaning stronger activation.

**Figure 4:**
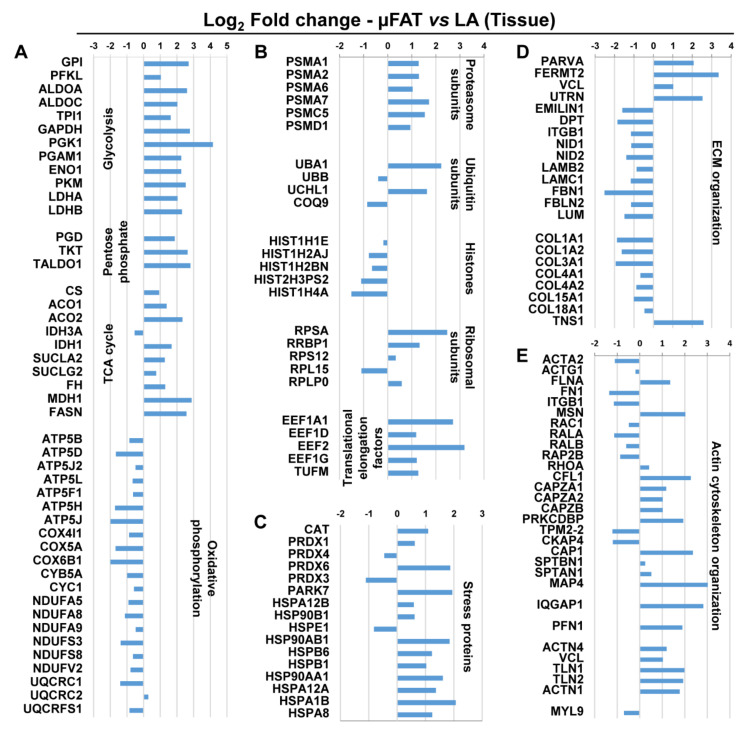
Modulated proteins in µFAT vs. LA tissues according to statistically relevant dysregulated pathways. All displayed proteins are significantly modulated (*p*-value < 0.05). (**A**) Metabolic proteins. (**B**) Structural proteins involved protein degradation and synthesis. (**C**) Stress-related proteins. (**D**) ECM organization including collagens. (**E**) Actin organization.

In the tissues (µFAT and LA), around 870 proteins were considered valid for quantification, and 544 proteins significantly (*p* ≤ 0.05) varied (Supplementary Table S1). The differently expressed tissue proteins were analyzed with Ingenuity Pathway Analysis (IPA) to identify dysregulated processes. The 5 most statistically significant pathways (according to *p*-value) are reported in Figure 3A (complete list in Supplementary Table S2). Notably, the first two pathways were related with mitochondria and oxidative stress, with the second one characterized by a negative z-score (−3.838) indicating the inhibition of the process in µFAT. A closer look at the proteins and pathways involved with cell metabolism revealed that proteins related to glycolysis, the pentose phosphate pathway and the TCA cycle were consistently upregulated in µFAT, as opposed to oxidative phosphorylation proteins (Figure 4A). Since alteration in cell homeostasis is often associated with protein turnover, a focused analysis also highlighted the up-regulation of proteasome and ubiquitin-related factors, translational elongation and ribosome-associated molecules, and down-regulations of histones (Figure 4B). Moreover, confirming the metabolic activation, chaperons also increased their expression (Figure 4C). Chaperones, and in particular members of the HSP70 and HSP90 families (HSPA1B, HSPA1A, HSPA12A, HSPA12B, HSP90AA1, HSP90AB1), are key players in mechanical stress protection. Eventually, as a response to microfragmentation and mechanical stress, a modulation of extracellular matrix, with reduction of collagens and adhesion-related proteins (Figure 4D), and actin cytoskeleton, through the activation of the talin-vinculin system (VCL, TLN1, TLN2) (Figure 4E), emerged.

**Figure 5:**
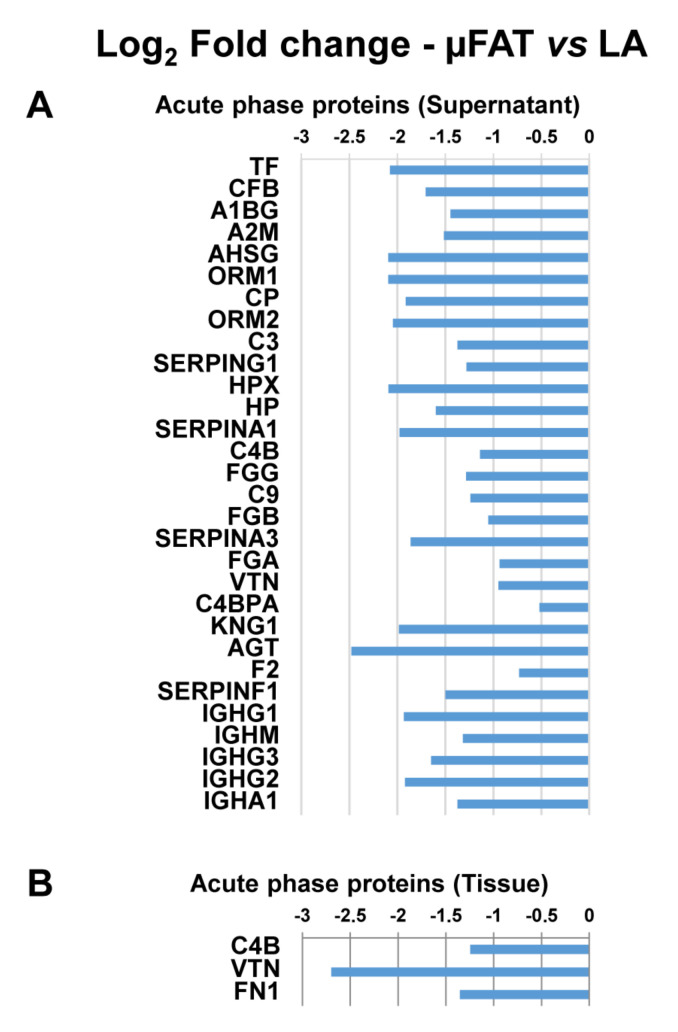
Modulated Acute phase proteins in µFAT vs. LA supernatants and comparison with tissues. All displayed proteins are significantly modulated (*p*-value < 0.05). (**A**) Supernatant. (**B**) Tissues.

In the supernatants, 287 proteins were considered valid for quantification, and 217 proteins significantly (*p* ≤ 0.05) varied between µFAT and LA (Supplementary Table S3). As with tissue proteins, IPA was used to identify dysregulated processes. The 5 most statistically significant pathways (according to *p*-value) are reported in Figure 3B (complete list in Supplementary Table S4). A large part of the categories appearing in the top positions of the ranking, such as Gluconoegenesis/Glycolysis (z-score = 2.646) and Ethanol degradation (z-score = 2.236), are related to cytosolic proteins and are present in the top positions of the tissues. With a different pattern, with respect to general cytosolic contamination leading to unbiased increment of proteins in µFAT samples, the Acute phase response pathway, defined by 30 proteins, appeared in the top positions with a negative z-score (−2.646), indicating the inhibition of the process in µFAT (Figure 5A). Notably, in the tissue only 3 proteins belonging to this pathway were found as dysregulated, confirming that supernatant data are free from cytosol contamination interference (Figure 5B).

### 3.5. Microfragmentation Allows for Reduction of Peripheral Blood-Derived EV-miRNAs and Increase of Chondro-Protective Ones

**Figure 6:**
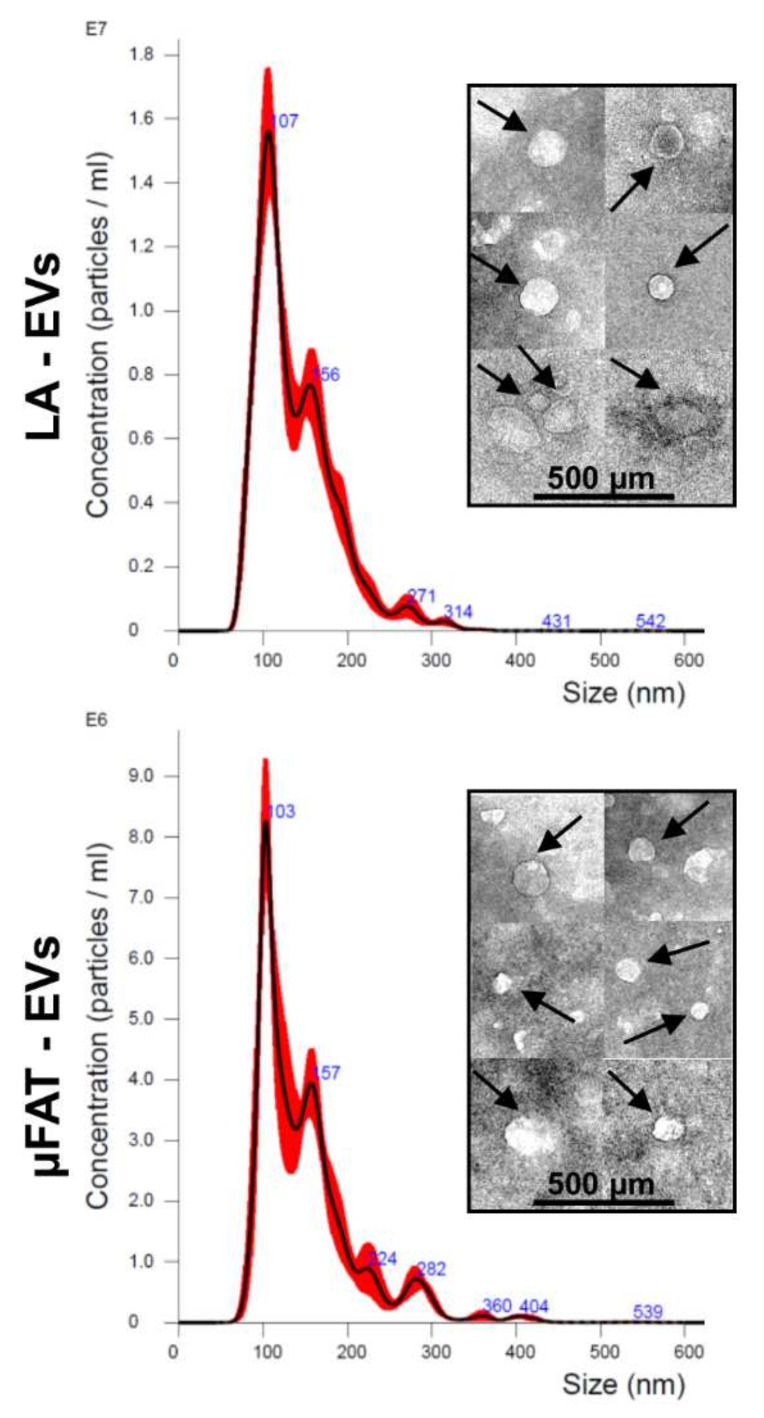
Phenotype of EVs released from µFAT and LA. NTA generated plots showing size distribution and of isolated particles. Images from a representative donor are shown. In the boxes, transmission electron micrographs of isolated EVs, as indicated by arrows.

Extracellular vesicles (EVs) released from LA and μFAT samples were analyzed by NTA (Figure 6). No significant (*p*-value ≤ 0.05) differences were observed, with mean particle size of 107.7 nm ± 16.8 for LA and 106.5 nm ± 7.7 for μFAT. TEM confirmed NTA dimensional range in isolated EVs. In total, 376 and 381 miRNAs were detected in LA and µFAT samples (Supplementary Table S5), with 57 significantly (*p* ≤ 0.05) varied between µFAT and LA and 5 newly gained. A more accurate search was focused on the most abundant ones falling in the first quartile of detection (Supplementary Table S6), that altogether contributed 98.5% and 98.6% of the total genetic message of LA and μFAT EV-miRNAs, respectively. Ninety-four miRNAs defined the first quartile for both LA and μFAT samples, for a total of 107 molecules. To make the analysis more stringent in view of unequivocal support for the removal of blood and blood-derived components previously observed in μFAT samples, a modulation ratio threshold of 5 (with a *p*-value ≤ 0.05) was applied. MiR-451a (μFAT vs. LA = 0.16 ± 0.07), highly abundant in EVs released by erythrocytes [30], and miR-486-5p (0.15 ± 0.09), related to EVs isolated from plasma [31], emerged. Of note in the frame of blood contamination reduction, although not laying in the first quartile of expression, the platelet-EV specific miR-223-5p [32,33] was strongly down-regulated (0.23 ± 0.05). Further, to confirm the presence of a stromal population enriched in ASCs, as demonstrated by flow cytometry, herein detected miRNAs were compared with those released from ASCs and previously identified with an identical technical approach from our laboratory [34]. Among the 5 most abundant miRNAs, 3 were shared (miR-193b-3p, miR-24-3p and miR-125b-5p) by LA, μFAT and ASCs. Altogether, these data corroborated the reduction of blood constituents and the maintenance of ASCs after tissue processing, as observed by flow cytometry.

Eventually, identified miRNAs were compared with those recently described to have a protective role in OA [35] and M2 anti-inflammatory macrophage polarization [36], to verify whether the EVs released by LA or μFAT contained potentially therapeutic molecules. In total, 34 miRNAs with OA protective functions were detected (Supplementary Table S7), with 17 in the first quartile of both LA and μFAT (Table 1). Scoring their levels, a 57% increase for the 17 most abundant miRNAs was observed in μFAT vs. LA-EVs, and this value increased up to 75% for those with a *p*-value ≤ 0.1. Regarding macrophage polarization, in total, 11 miRNAs with M2 anti-inflammatory promoting features were detected (Supplementary Table S8), with 7 in the first quartile of both LA and μFAT (Table 2). Analyzing their modulation, a 29% increment for the most abundant ones emerged in μFAT vs. LA-EVs. Of note, miR-181a-5p, that showed the highest and most significant fold-change (μFAT vs. LA of 4.30 ± 0.66 with *p*-value of 0.038), resulted in only µFAT-EVs in the first quartile.

## 4. Discussion

In this report, a deep characterization of donor-matched LA and μFAT showed that microfragmentation does not alter adipose tissue architecture and stromal cell content, while it provides clearance of blood-derived elements. Moreover, proteomic analysis and EV-embedded miRNA quantification highlighted both the reduction of blood-derived, and pro-inflammatory, miRNAs, and an increase in cartilage-protective factors.

Microfragmentation was shown to produce smaller tissue pieces with respect to classical lipoaspiration using the Coleman technique, thus increasing the surface/volume ratio compared with larger tissue pieces [14]. Consistently, in the same study, it was reported that ASC migrated more easily from μFAT lobules than in conventional LA. Also, smaller lobules allowed for increased cell viability, possibly due to enhanced oxygen supply via shorter diffusion. This could be of crucial importance, since, in a clinical setting, higher viability and migration rate, and reduced depth, allowing for quicker release of secreted factors to the graft surface, might provide more beneficial effects for patients. In this frame, to be compliant with the minimally manipulated products definition, the processing technique does not have to alter the architecture and the composition of active cell populations. Our histology results clearly showed that μFAT and LA tissues are similar in overall architecture, including the presence of small vessels and related CD31^+^ endothelial cells. Nevertheless, the presence of blood-derived cells, such as erythrocytes drastically dropped, thereby reducing the pre-inflammatory actions attributed to these elements in the joint microenvironment. These data were further confirmed by cell count and flow cytometry data for mononuclear cells, platelets and erythrocytes, confirming that processing, through washing steps, allows for efficient removal of blood contaminants maintaining overall structure, including vessels, intact. Of note, flow cytometry data also confirmed the conserved presence of CD31^+^ endothelial cells between μFAT and LA. Of crucial importance for μFAT paracrine activity, both products had a similar content of active stromal cells, such as ASC and pericytes. Importantly, correlation analyses for the identified stromal cell types confirmed that, overall, the microfragmentation procedure does not significantly alter stromal cell ratios compared to LA. Taken together, these data clearly indicate that microfragmentation does not select for specific cell subpopulations and results in tissue with identical architectural and stromal composition, when compared to unprocessed LA.

Microfragmentation was able to produce changes, in terms of both tissue and released factors, if there was overall similar architecture and stromal cell composition. It clearly emerged that processing, as expected during tissue disruption, brought some stress and cell lysis as evidenced from reduction in cell viability and release of cytosolic proteins in the supernatant, as well as the detection of protein turnover-related factors and chaperones in the tissues. Moreover, proteomic data showed the attempt of microfragmented tissue to counteract tissue loosening by activating cell metabolism at different levels. Notably, acute phase factors significantly reduced in µFAT secretome. Acute phase proteins are a reaction to tissue injury, including the damaged joint, and involve inflammation [37]. In particular, acute phase reactants have been detected in synovial fluids from OA patients [38] and their levels are both a target for therapy and an indicator of disease progression [39,40]. Both cytokines and chemokines are elevated in OA patients undergoing surgery [41] and have been identified in synovial tissues in both early and late OA [42,43]. Within acute phase factors, A1/A3/F1/G1 Serpins were found to be downregulated in µFAT. These factors are especially gaining interest for the development and onset of OA pathology [44], as they, overall, regulate the proteases involved in the degradation of ECM. Nevertheless, due to the elevated number of factors and contrasting activities, it is not easy to predict a net effect. In fact, SerpinA1/3 inhibits neutrophil serine proteinases, including neutrophil elastase and proteinase-3 and cathepsin G, respectively [45]. Emerging evidence suggests that neutrophil elastase has a role in OA [46], meaning changes in levels of regulating serpins might be of particular importance. Regarding SerpinF1, by binding to ECM components, it has catabolic activity and promotes cartilage destruction, together with being reported to increase in OA cartilage [38]. SerpinG1, or C1 inhibitor, participates in the activation of the complement system [47], the dysregulation of which in synovial joints contributes to inflammation and plays a critical role in OA pathogenesis [48]. Together with SerpinG1, several other complement-related factors (CFB, CP, C3, C4B, C4BPA, C9) were downregulated in µFAT. Eventually, the coagulation system also emerged as a significantly regulated pathway (Supplementary Table S4). Activation of the coagulation cascade in the joint is crucial in both inflammatory and degenerative joint diseases [49]. In particular, F2 encoding for thrombin and FGB, that is important for blood clot formation, were downregulated in µFAT. Therefore, overall, reduced levels of these factors in μFAT might be beneficial, although we are aware that the contamination of cytosolic factors in the supernatant is a bias of the presented results.

Adipose tissue EVs have been postulated as important mediators for the regenerative features of fat in the management of several diseases, including orthopaedic pathologies. In particular, miRNAs were the active players behind EVs’ action [50], and, therefore, we focused our attention on their fingerprint in LA and μFAT EV-samples. In this frame, the sharp decrease of miR-451a (enriched in erythrocytes-EVs) [30], miR-486-5p (abundant in plasma–EVs) [31] and miR-223-5p (strongly present in platelets-EVs) [32,33] confirmed the efficient removal of blood and its components. Yet, the high correspondence between LA and μFAT EV samples, observed by flow cytometry, might be due to the maintenance, even after processing, of the main cell populations that are able to secrete extracellular vesicles, and associated miRNAs, such as mesenchymal stem cells (MSCs) [26,34]. Consistently, the presence of miR-193b-5p, miR-24-3p and miR-125b-5p among the most 5 expressed EV-miRNAs in LA, μFAT and ASCs EV-samples confirmed that the ASC population was strongly present and maintained in LA and μFAT, and that its secreted vesicles predominantly contributed to the overall abundance of the herein detected miRNAs. Furthermore, the high contribution of miR-125b-5p by ASCs [34] explains why this miRNA, highly present in the EVs of erythrocytes [30], was not found decreased in μFAT samples following removal of the blood component, unlike miR-451a and miR-486-5p, which in EVs from ASCs are found at extremely low levels, or miR-223-5p, which is not present at all.

To substantiate the capacity of adipose tissue to counteract joint diseases, such as OA, miRNAs reported to have protective functions in OA [35] were sifted and 34 were detected. For the 17 that were highly expressed in both LA and μFAT EVs, and lay in the first quartile of expression, an average 1.5 increase in the overall quantity was observed. Intriguingly, the highly abundant and ASC EVs-specific miR-193b-5p and miR-24-3p, although not reaching statistical significance, were upregulated in all analyzed µFAT samples, with each sample having at least one of the two increasing atby a factor ≥ 2. miR-193b-5p has been shown to have a direct role in controlling metabolism in inflamed chondrocytes by reducing the expression of *MMP3* and *MMP13* metalloproteases [51]. miR-24-3p, similarly, has a protective role by reducing senescence and catabolism of damaged cartilage [52]. It should also be noted that the 2 miRNAs that are greatly reduced in μFAT-EVs, miR-451a and miR-486-5p, increased in expression in the cartilage of patients with osteoarthritis [53,54]. Therefore, their decrease after microfragmentation could further increase the protective capabilities of μFAT. Eventually, 11 miRNAs with pro-M2 macrophage polarizing features were identified, and 7 were among the most abundantly encapsulated in EVs. As for OA-protective molecules, microfragmentation lead to an average increase (1.3 fold), and miR-24-3p again led the modulation. In particular, miR-24-3p was reported to both repress M1 inflammatory, and promote M2 anti-inflammatory, phenotypes [55]. A similar behavior was described for miR-181a-5p [56], which showed the highest modulation (4.30 fold) in µFAT-EVs being included in the first quartile of abundance. Therefore, it can be concluded that EVs secreted by LA or μFAT contain several cartilage-protecting and M2 anti-inflammatory macrophage miRNAs, and that the increased presence of protective EV-miRNAs after microfragmentation may support the hypothesis of the superior protective and anti-inflammatory features of μFAT.

These data, together with the soluble factors fingerprint, give molecular grounds for μFAT features reported in several models related to OA. In primary human chondrocytes, μFAT-induced cell proliferation, ECM synthesis and glycosaminoglycan level increased compared to LA [57]. In rat chondrocytes, μFAT significantly stimulated cell migration in wound areas, with cartilage defects displaying regular surface, a high amount of hyaline cartilage, intact subchondral bone reconstruction and corresponding formation of type I, II, and VI collagen, which resembled normal cartilage [58]. In synovia derived cells, including both fibroblasts and macrophages, μFAT supported cell proliferation and had anti-inflammatory effects, including the reduction of both synovial macrophage chemokines and cartilage matrix degrading enzymes [59]. Finally, in LPS-stimulated macrophages μFAT showed strong anti-inflammatory capacity by attenuating pro-inflammatory cytokine release [60].

Together with the relevant strength of comparing LA and µFAT obtained from the same patients and the use of cutting-edge technologies for secretome fingerprinting, we are aware that this study has limitations. First, donors of the study were subjected to liposuction for aesthetic procedures and not affected by musculoskeletal disorders, such as OA, as normally happens for patients undergoing orthobiologics- (including µFAT) based procedures. However, this decision was based on the need for sufficient µFAT allowing for all the analyses. Second, qRT-PCR-based miRNA detection relies on a reduced number of miRNAs that are rapidly increasing in their overall number. We preferred to use this solid technology, due to both the deep overall characterization and the bunch of OA-related data reported for the herein sifted molecules. Therefore, in the future, a larger number of miRNAs will have to be scored, based on available new literature. Third, the use of serum-free media for secretome collection is far from the OA environment, albeit, to date, culturing conditions for secretome analysis that avoid serum interference and recapitulating OA joints are still far from being defined. Again, future studies will be fundamental when OA-recapitulating conditions become clearly defined by clinical and scientific communities.

## 5. Conclusions

In conclusion, microfragmented adipose tissue presents intact architecture and stromal cell composition with respect to LA, together with a reduced amount of blood elements that might be detrimental for the joint microenvironment. Also, μFAT showed a molecular signature enriched in anti-inflammatory and chondroprotective mediators. Taken together, cell and molecular data suggest that μFAT is a potent tool for management of joint degeneration, by addressing the main molecular pathways involved in this disorder. Whether μFAT is able to provide joint tissue regeneration, in addition to addressing pain and inflammation, has yet to be proven. 

## Figures and Tables

**Table 1:** Chondroprotective miRNAs in LA-EVs and µFAT-EVs first quartile.

miRNA	µFAT vs. LA	Role
miR-193b-5p	1.90	Inhibit degradation of ECM components and moderate inflammation
miR-24-3p	1.93	Inhibit senescence, cartilage catabolism and chondrocyte apoptosis
miR-320a-3p	0.43	Promote chondrocyte viability and chondrogenesis
miR-92a-3p	0.61	Inhibit cartilage catabolism and promote collagen deposition
miR-222-3p	0.73	Inhibit cartilage degradation
miR-17-5p	1.03	Induce autophagy
miR-30a-5p	1.28	Promote cartilage homeostasis
miR-152-3p	1.03	Decrease synovial fibroblast proliferation
miR-199a-3p	1.74	Anti-catabolic
miR-130a-3p	0.89	Anti-inflammatory
miR-210-3p	0.73	Inhibit apoptosis and promote chondrocyte proliferation and ECM deposition
miR-26a-5p	3.32	Promote cartilage homeostasis
miR-29a-3p	3.63	Inhibit excessive cartilage remodeling
miR-27a-3p	1.59	Prevent synovial fibroblast migration and invasion
miR-148a-3p	1.56	Promote hyaline cartilage production
miR-26b-5p	2.55	Promote cartilage homeostasis
miR-27b-3p	1.72	Anti-catabolic

LA, lipoaspirate; EV, extracellular vesicles; μFAT, microfragmented adipose tissue; ECM, extracellular matrix.

**Table 2:** Pro M2 anti-inflammatory macrophage miRNAs in LA-EVs and µFAT-EVs first quartile.

miRNA	µFAT vs. LA	Role
miR-24-3p	1.93	Anti M1—Pro M2
miR-30d-5p	1.03	Anti M1—Pro M2
miR-146a-5p	0.55	Anti M1—Pro M2
miR-146b-5p	2.34	Anti M1—Pro M2
miR-34a-5p	0.84	Pro M2
miR-222-3p	0.73	Pro M2
let-7b-5p	1.59	Pro M2

M1, macrophage type 1; M2, macrophage type 2.

## Data Availability

Data is contained within the article or Supplementary Materials.

## Supplementary Materials

The following are available online at https://www.mdpi.com/article/10.3390/jcm11082231/s1. Table S1: Detected proteins and differential expression in µFAT vs. LA tissues; Table S2: Statistically altered pathways in µFAT vs. LA tissues; Table S3: Detected proteins and differential expression in µFAT vs. LA supernatants; Table S4: Statistically altered pathways in µFAT vs. LA supernatants; Table S5: Detected EV-miRNAs (normalized C_RT_ values); Table S6: EV-miRNAs falling in the first quartile of abundance for LA and µFAT; Table S7: Chondroprotective miRNAs in LA-EVs and µFAT-EVs; Table S8: Chondroprotective miRNAs in LA-EVs and µFAT-EVsr; Figure S1: Flow cytometry gating strategy to identify vascular endothelial cells (VEC), adipose mesenchymal stromal cells (ASC) and pericytes (PER); Figure S2: Representative micrographs of osteogenic, adipogenic and chondrogenic differentiation of ASCs cultured after isolation from LA and μFAT. Scale bar 200 μm.
