# Supplementary material for: Characterization of Microfragmented Adipose Tissue Architecture, Mesenchymal Stromal Cell Content and Release of Paracrine Mediators
Source: J Clin Med. 2022 Apr 15;11(8):2231. doi: 10.3390/jcm11082231 (PMC9026471; doi:10.3390/jcm11082231)
Supplement: Supplementary file 1 [file jcm-11-02231-s001.zip › Supplementary Figures 1 and 2_JCM.pdf]

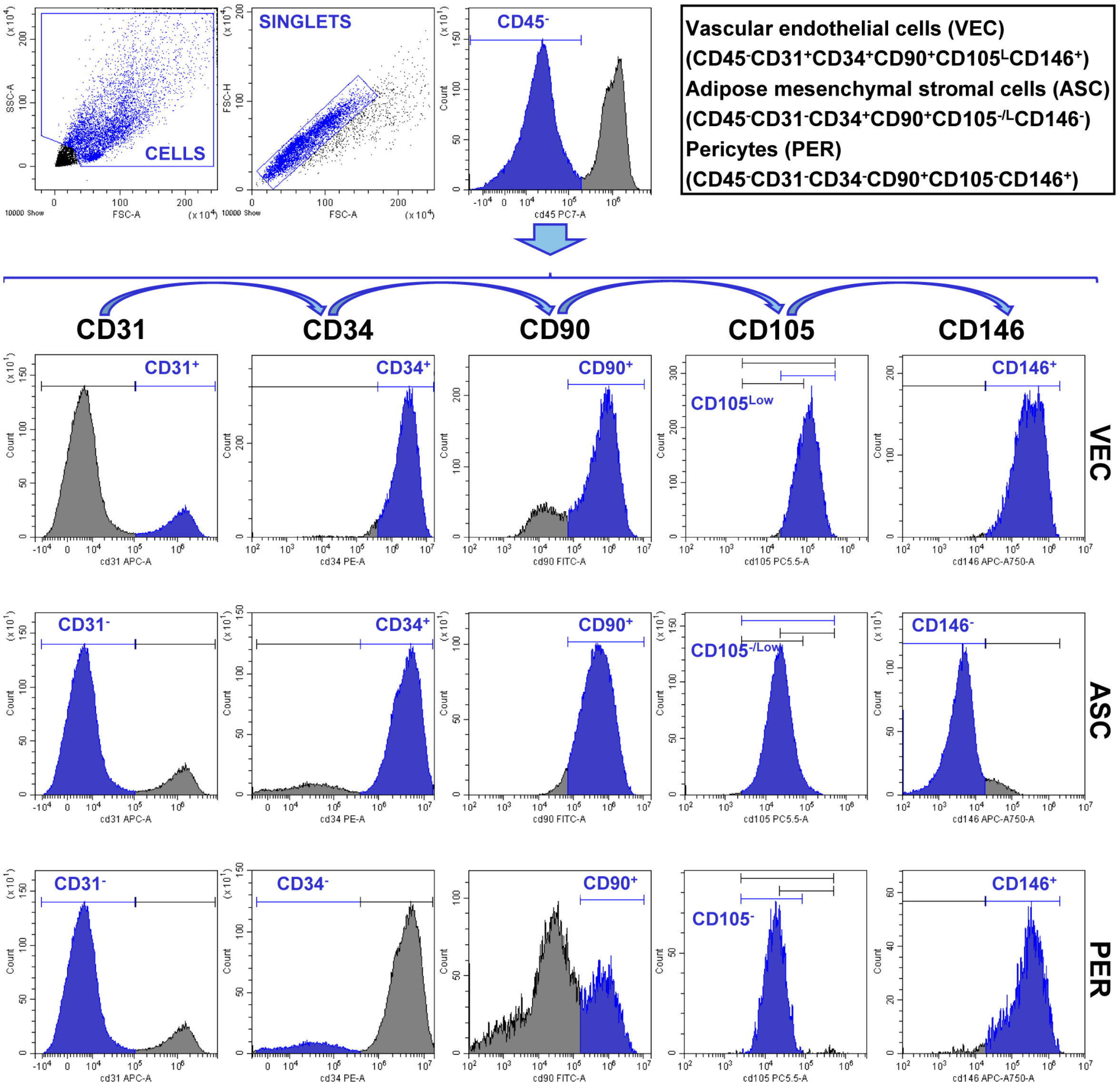

Figure S1. Flow cytometry gating strategy to identify vascular endothelial cells (VEC), adipose mesenchymal stromal cells (ASC) and pericytes (PER).

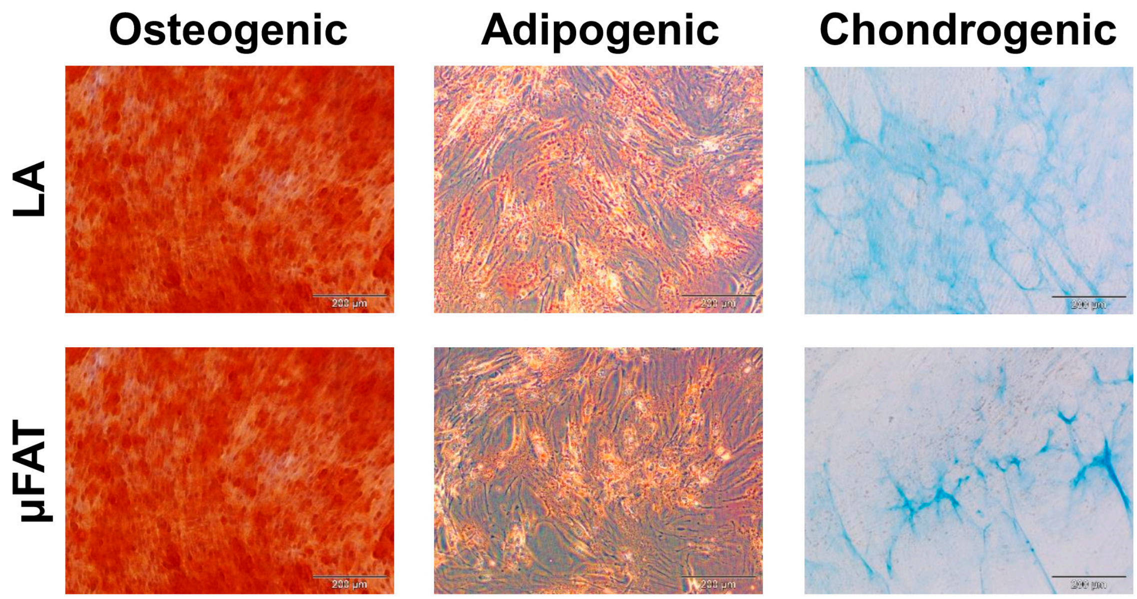

Figure S2. Representative micrographs of osteogenic, adipogenic and chondrogenic differentiation of ASCs cultured after isolation from LA and μFAT. Scale bar 200 μm.
